# Evolutionary Analysis and Catalytic Function of LOG Proteins in Plants

**DOI:** 10.3390/genes15111420

**Published:** 2024-10-31

**Authors:** Chunjie Zhao, Huanran Yin, Yuqi Li, Jiacheng Zhou, Siteng Bi, Wenhao Yan, Yunzhen Li

**Affiliations:** National Key Laboratory of Crop Genetic Improvement, Hubei Hongshan Laboratory, Huazhong Agricultural University, Wuhan 430070, China

**Keywords:** LOG, motif, catalytic activity, cytokinin, plant

## Abstract

Background: The plant hormone cytokinin is a conserved regulator of plant development. LONELY GUY (LOG) proteins are pivotal in cytokinin biosynthesis. However, their origin, evolutionary history, and enzymatic characteristics remain largely uncharacterized. Methods: To elucidate LOG family evolution history and protein motif composition, we conducted phylogenetic and motif analyses encompassing representative species across the whole green plant lineage. Catalytic activity and structure analysis were conducted to thoroughly characterize the LOG proteins. Results: Our phylogeny showed that LOG proteins could be divided into five groups and revealed three major duplication events giving rise to four distinct groups of vascular LOG proteins. LOG proteins share a conserved structure characterized by a canonical motif arrangement comprising motifs 1, 2, 3, 4, 5, 6, and 7. Two significant changes in LOG motif composition occurred during the transition to land plants: the emergence of motif 3 in charophyte LOG sequences and the subsequent acquisition of motif 8 at the C-terminus of LOG proteins. Enzymatic assays demonstrated that LOG proteins can be classified into two groups based on their enzyme activity. One group act as cytokinin riboside 5′-monophosphate phosphoribohydrolase and primarily convert iPRMP to iP, while the other group act as 5′-ribonucleotide phosphohydrolase, and preferentially produce iPR from the same substrates. TaLOG5-4A1, TaLOG5-4A2, TaLOG5-5B2, and TaLOG5-D1 shared conserved residues in the critical motif and were predicted to have similar protein structures, but displayed distinct enzymatic activities. Conclusions: Our findings provide a comprehensive overview of LOG protein phylogeny and lay a foundation for further investigations into their functional diversification.

## 1. Introduction

Cytokinins are key plant hormones that regulate a wide range of developmental processes, including cell division, shoot and root growth, abiotic and biotic stress responses, nitrogen-responsive growth promotion and leaf senescence [1,2,3,4,5,6,7,8,9]. The spatial and temporal expression of *LOG* genes contributes to the precise regulation of cytokinin levels and plant growth and development, including cell division, vascular development, apical dominance, and leaf senescence [10,11]. Disruption of LOG function leads to pleiotropic developmental defects, highlighting the essential role of LOG proteins in plant growth and morphogenesis [12].

De novo cytokinin biosynthesis commences with the formation of the N^6^-(Δ^2^-isopentenyl) adenine (iP) riboside 5′-tri-, di- or monophosphate (iPRTP, iPRDP or iPRMP, respectively) [13,14,15]. In addition, cis-zeatin riboside monophosphate (cZRMP), derived from the degradation of prenyl-tRNA, is also an important precursor [16]. These products undergo subsequent hydroxylation at the prenyl side chain terminus by CYP735A to yield the trans-zeatin (tZ) riboside 5′-tri-, di- or mono-phosphate (tZRTP, tZRDP or tZRMP, respectively) [17]. Subsequently, two pathways lead to the generation of cytokinin free bases, which are the bioactive form of cytokinin. In the one-step pathway, the activation of ribonucleotides to generate free bases is catalyzed by 5′-monophosphate phosphoribohydrolases encoded by *LONELY GUY* (*LOG*) genes [4,14,18]. In the two-step pathway, 5’-ribonucleotide phosphohydrolase first converts the compound into ribonucleosides. Then, adenosine nucleosidase catalyzes the formation of the active form of cytokinin. Conversion of the hydrolysis of cytokinin nucleosides to free bases can be catalyzed by N-ribohydrolases (NRHs), identified in both *Physcomitrium patens* and *Zea mays* [19], and the cytokinin/purine riboside nucleosidase 1 (CPN1), identified in rice (*Oryza sativa* L.) [20]. The most recent study has found that a rice LOG protein, Grain yield 3(GY3)/OsLOGL5, exhibits as 5′-ribonucleotide phosphohydrolase converting iPRMP to iP riboside (iPR) [7], which expands the role of LOG proteins. Whether this ribonucleotide phosphohydrolase activity is evolutionarily conserved in other species (e.g., *Triticum aestivum*) has not been investigated so far, but this knowledge is important for understanding cytokinin biosynthesis in plants.

LOG homologs were found in several green algae species, but with only one single copy in each genome [21]. Biochemical investigation of *Chlorella variabilis* LOG (CvarLOG) showed that it exhibits similar enzymatic activity in the conversion of iPRMP and tZRMP to the free-base forms iP and tZ, respectively [22]. Phylogenetic analysis with LOG protein sequences from several species revealed a tree comprising two clades, with clade I coming exclusively from seed plants [22]. Besides this, the evolutionary history of LOG across plant lineages is largely unknown so far.

This study aims to elucidate the evolutionary trajectory of LOG proteins by conducting a comparative analysis of *LOG* gene sequences across diverse plant lineages. By reconstructing the phylogenetic relationships among LOG proteins and investigating their structural and functional divergence, we aim to identify key evolutionary events that shaped the LOG protein family. Additionally, we explore the correlation between the evolutionary changes in LOG protein sequences and their catalytic functions.

## 2. Material and Methods

### 2.1. Data Collection

The protein sequences for each plant species were obtained from the Joint Genome Institute (JGI) website (http://genome.jgi-psf.org/, accessed on 21 July 2023) and National Center for Biotechnology Information (NCBI). LOG protein sequences were identified in plant species with complete genome sequences by using the protein-to-protein Basic Local Alignment Search Tool (BLASTP) and Hidden Markov Model (HMM) search methods. The obtained LOG sequences were further validated on the InterPro website (https://www.ebi.ac.uk/interpro/, accessed on 30 July 2024) to confirm the existence of the PTHR31223 domain based on protein analysis through evolutionary Relationships (PANTHER) annotations.

### 2.2. Phylogenetic and Motif Analysis

Amino acids sequences were aligned with Multiple Alignment of Amino Acid or Nucleic Acid Sequences (MAFFT) [23], and trimmed with trimAl [24] using the “-cons 0.0 -gt 0.8 -st 0.0” command. Maximum-likelihood phylogenies were inferred using IQ-TREE [25] under the JTT + R7 model for 20,000 ultrafast [26] bootstraps. The visualization of the phylogenetic tree was conducted online using the tool iTOL (https://itol.embl.de/, accessed on 11 May 2024). Motif analysis was conducted with Multiple EM for Motif Elicitation (MEME) search and visualization by using the TBtools software [27]. For critical residue analysis, multiple-protein-sequence alignment was conducted with MAFFT [23] and visualized using the online tool ESPript [28].

### 2.3. Plasmid Construction and Protein Expression

The coding sequences of *TaLOG* genes were amplified from cDNA by using primers in Table S1. The amplified sequences were inserted into the pET-28 vector by using the Gibson Assembly method [29]. The confirmed vectors were transformed into an *Escherichia coli* (*E*. *coli*) BL21 (DE3) strain to express the LOG proteins under the induction of 1 mM isopropyl-d-thiogalactopyranoside (IPTG) in LB media for 16 h at 16 °C. LOG proteins were conducted by using Ni-NTA Agarose Resin (Thermo Fisher Scientific, Rockford, USA) according to the manufacturers’ protocol.

### 2.4. Enzymatic Assays

Enzymatic analysis was conducted as described in [7] with some modifications. Enzymatic activity was measured by incubating the samples with the same amount (0.78 μg) of each recombinant purified LOG protein in a reaction mixture consisting of 50 mM Tris-HCl, 1 mM MgCl_2_ and 1 mM dithiothreitol (DTT) (pH 6.5) with the substrate iPRMP at 30 °C for 2 h. All the reaction products were purified through an Oasis MCX column (Waters Corporation, Milford, MA, USA) and measured with an LC/MS system (Agilent 6520 Accurate-Mass Q-TOF LC/MS, Santa Clara, California, USA; Agilent Zorbax C18, 4.6 mm 350 mm, Santa Clara, CA, USA) under the conditions described before [30].

### 2.5. Prediction of Protein Structure

Protein sequences were uploaded into the AlphaFold server (https://alphafoldserver.com/, accessed on 13 October 2024) to produce the structure with default settings. The predicted protein structure outputs were downloaded and were subjected to pairwise structure alignment with the online tool provided by the Protein Data Base (https://www.rcsb.org/alignment, accessed on 13 October 2024) to compare the protein structures. The Root Mean Square Deviation (RMSD) and Template Modeling (TM) score were used for the evaluation of the similarity of structures of proteins of interest.

## 3. Results

### 3.1. Phylogenetic Distribution Pattern of LONELY GUY (LOG) Gene in Green Plants

**Figure 1:**
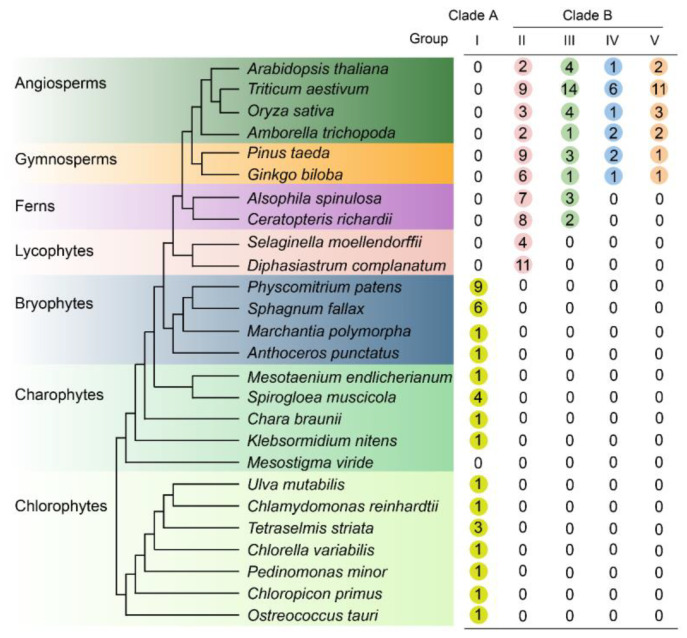
Distribution of *LONEY GUY (LOG)* genes across the green plants. The order of tree branches and the divergence time are derived from Phytozome (https://phytozome-next.jgi.doe.gov/, accessed on 21 July 2023) with some modifications. LOG homologs were identified in each plant genome by using the protein-to-protein Basic Local Alignment Search Tool (BLASTP) and Hidden Markov Model (HMM) search methods. LOG proteins belong to family PTHR31223 based on protein analysis through evolutionary relationships (PANTHER) annotations were used for analysis. The number of different LOG proteins was counted according to the phylogenic relationship and are listed in the table on the right side of the species tree.

To investigate the distribution pattern of LOG proteins, representative species across the plant kingdom were selected for the analysis. These species included green algae (including chlorophytes and charophytes), early land nonvascular plants (i.e., liverworts, hornworts and mosses), spore-producing vascular plants (i.e., lycophytes and ferns), and vascular seed plants (i.e., gymnosperms and angiosperms). LOG homologs were identified in each plant genome using both BLAST and HMM methods. The initial analysis of the HMM search against the Pfam database with the identifier PF03641 yielded two clearly separated clades (clade *α* and *β*) in the phylogenetic tree (Figure S1). All the reported LOG homologs were in clade *β*. Both clades retain independent potential ancestor sequences from the chlorophytes, which suggests that they are two different protein families. Therefore, clade *β*, belonging to the LOG protein family (YJL055W, PTHR31223) based on PANTHER annotations, was used for further investigation (Table S2, Figure 1).

**Figure 2:**
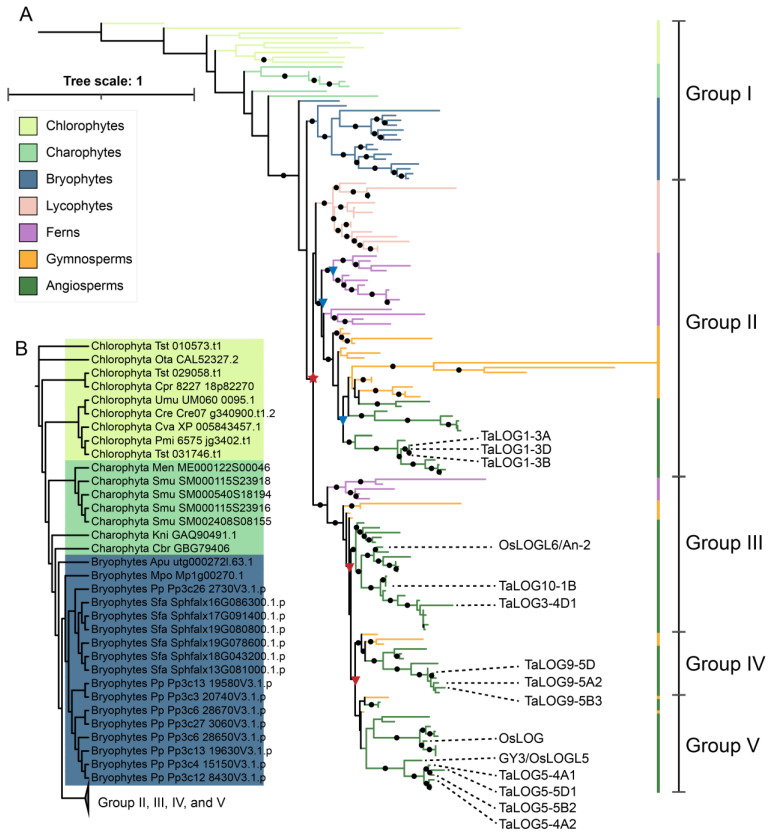
Phylogenetic analysis of the LOG family across the green plants. (**A**) Maximum-likelihood phylogenies were inferred using IQ-TREE [25] under the JTT+R7 model for 20,000 ultrafast [26] bootstraps. Black dots indicate support values of bootstrap analyses > 90. The scale bar indicates the number of changes per site. Duplication events are indicated with a star (tandem duplication, TD) and triangle (whole-genome duplication, WGD). Three major duplication events are highlighted in red. (**B**) A detailed phylogenetic relationship between green algae and early land bryophytes is listed with each protein ID.

Maximum likelihood phylogenetic analysis was further conducted with an alignment composed of all LOG members. The obtained tree clearly showed that the LOG sequences were split into two main clades: clade A (including only group I) and clade B (including groups II, III, IV and V) (Figure 2A). Clade A contains sequences from green algae and early land nonvascular plants, which represent the ancestor sequences. Sequences in clade B are all from vascular plants, forming a monophyletic branch.

### 3.2. Origin and Expansion of LOG Family

The phylogenetic tree indicated that the ancestral *LOG* gene originated from chlorophyte algae. Most chlorophyte algae possess a single LOG homolog, with the exception of *Tetraselmis striata*, which contains three LOGs (Figure 1 and Figure 2B). Among the charophytes, *Spirogloea muscicola* harbors four LOG members, while the remaining species possess only one. No LOG homolog was detected in *Mesostigma viride*. In bryophytes, a single LOG was identified in both hornworts and liverworts (Figure 1 and Figure 2B). In contrast, mosses exhibited a significant expansion of the LOG family, with nine and six members found in *P. patens* and *Sphagnum fallax*, respectively. Subsequent plant lineages consistently contained at least four LOG members. These results indicated that all land plant LOGs evolved from a single gene already present in chlorophyte algae.

**Figure 3:**
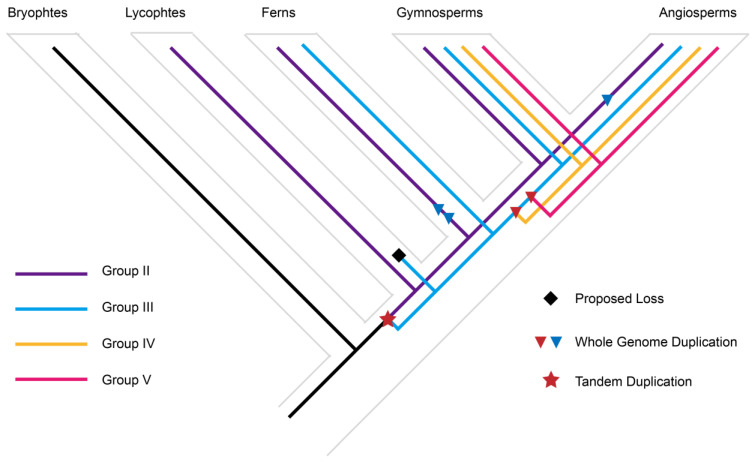
Duplication events in the LOG protein family inferred from protein-level phylogenetic analyses. Schematic depicting the inferred history of gene duplication and loss within green plants. Inferred duplications are marked with a star (TD) and triangles (WGDs), and proposed losses are indicated with black diamonds. Three major duplications are highlighted in red.

One notable result of our phylogenetic analysis is that the bryophyte and vascular LOGs together form a monophyletic group. One of the bryophyte sequences (Bryophytes_Apu_utg000272l.63.1 in Figure 2A, Table S2) is divergent from other bryophyte sequences, and thus its phylogenetic position is not well supported in the tree. The rest of the bryophyte LOGs form a lineage that is orthologous to all vascular plant lineages (Figure 2A). Three major duplication events were inferred from the evolution history of LOGs. One tandem duplication event occurred before the diversification of vascular plants to give rise to group II and a large clade composed of group III, IV and V. Another two whole-genome duplications occurred before the diversification of ferns to generate group III, IV and V (Figure 3). Group III is one of the earliest-diverging LOG lineages, and is composed of LOG sequences from lycophytes, ferns, gymnosperms, and angiosperms. However, the LOG sequence from the lycophyte sequence is absent from the sister lineage, group III. Thus, we inferred lycophyte loss from group III (Figure 3).

### 3.3. The Evolutionary Changes in Conserved Motifs in LOG Homologs

**Figure 4:**
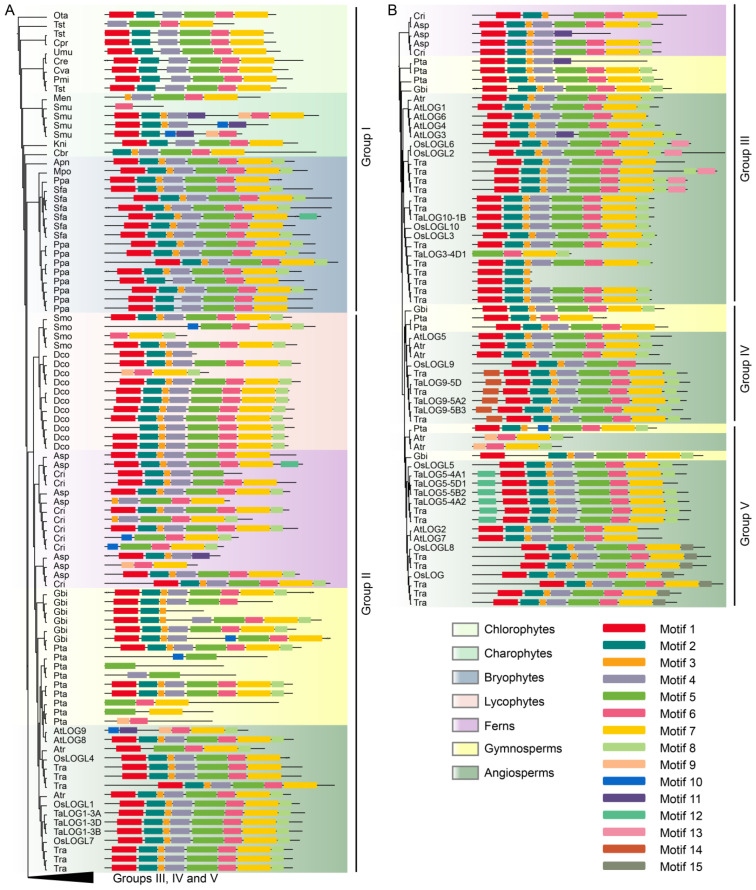
Alterations in protein motif structure of LOGs during evolution. (**A**) Different colors of rectangles indicate different motifs. The phylogenetic relationship of LOG proteins is shown on the left side of the motif structure schematics. The black triangle represents the collapsed phylogenetic tree including group III, IV and V. The collapsed phylogenetic tree along with the motif structure are shown in (**B**).

To elucidate the evolutionary trajectory of LOG homologs, we conducted a comparative analysis using their protein sequences. Our findings revealed that LOG homologs shared conserved motifs across the green plant lineage. Notably, ten of the sixteen ancestral LOG copies from chlorophytes contained motifs 1, 2, 4, 5, 6, and 7, which were also present in LOG homologs of more derived plant groups (Figure 4A). In contrast, before the colonization of land by plants, charophyte LOG sequences exhibited the most diverse motif composition. These include the initial emergence of motifs 3, 9, 10, and 11, as well as truncations of certain motifs, particularly notable in charophyte *S. muscicola* (*Smu*). The newly evolved charophyte motif 3 was retained in more derived plant groups. Conversely, motifs 9 and 10 were shared exclusively by lycophytes, ferns, gymnosperms, and the basal angiosperm *Amborella trichopoda*, but were completely lost in more derived angiosperm species (Figure 4A,B). A canonical LOG structure comprising motifs 1, 2, 3, 4, 5, 6, and 7 became the predominant LOG form from the bryophytes onward. Our analysis also revealed that motif 8 was first acquired by bryophyte LOG sequences at the C-terminus and subsequently retained in descendant lineages. Based on phylogenetic relationships, we divided angiosperm LOGs into four groups (i.e., groups II, III, IV, and V). Motif structure analysis revealed that all angiosperm LOGs shared a similar composition and order of motifs. However, novel motifs emerged in certain groups: motif 13 in group III, motif 14 in group IV, and motif 12 and motif 15 in group V. In summary, our results demonstrate that LOG proteins exhibited a conserved motif structure across green plants, which might have contributed to the colonization of land by plants and the subsequent dominance of angiosperms.

### 3.4. The Catalytic Activity of Different LOG Proteins

**Figure 5:**
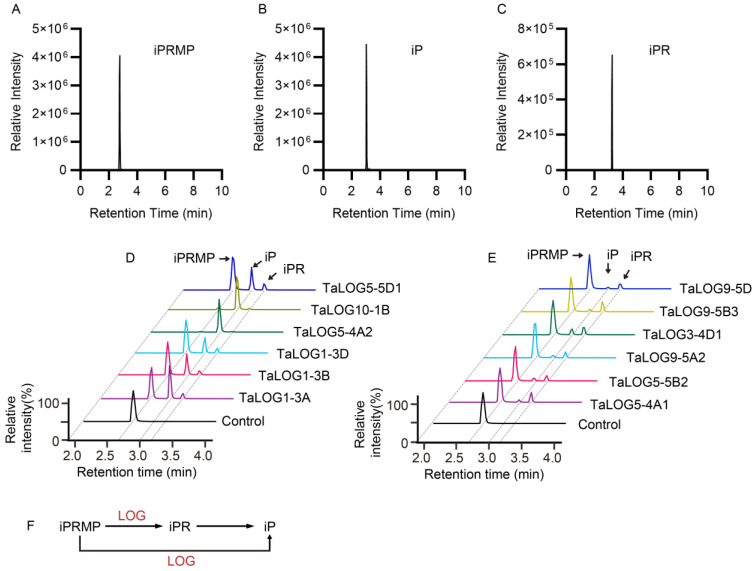
Enzymatic analysis of LOG proteins. (**A**–**C**) Peaks of N^6^-(Δ^2^-isopentenyl) adenine riboside 5′-monophosphate (iPRMP) (**A**), N^6^-(Δ^2^-isopentenyl) adenine (iP) (**B**), and iP riboside (iPR) (**C**). (**D**,**E**) Products of indicated LOG proteins and control reacted with substrate iPRMP. (**F**) Schematic representation of involvement of LOG proteins in the cytokinin biosynthesis and activation pathway.

A recent study found that GY3/OsLOGL5 functioned in the two-step pathway to produce cytokinin in rice (*O. sativa* L.) rather than the one-step pathway [7,14]. We speculated that the phylogenetic relationship of LOG proteins may correlate with their catalytic function character. To clarify this, we selected different LOG proteins according to their phylogenetic classification (Figure 2A and Figure 4A,B, Table 1). These LOG proteins were expressed in *E*. *coli* and were purified for enzymatic activity assays. In vitro reactions were conducted using equal amounts of N^6^-(Δ^2^-isopentenyl) adenine riboside 5′-monophosphate (iPRMP) as a substrate. Reaction products were analyzed by a liquid chromatography–mass spectrometry (LC/MS) system. Our results indicated that all tested LOG proteins catalyzed the conversion of iPRMP (Figure 5A) to both iP (Figure 5B) and iPR (Figure 5C). However, significant variations in product ratios were observed among LOG proteins (Figure 5D–E, Table 1). Based on product distribution, LOG proteins were categorized into two groups: the iP group, primarily producing iP with an iPR/iP ratio less than 1, and the iPR group, predominantly generating iPR with iPR/iP ratios greater than 1. In particular, both the substrate iPRMP and the product iPR were present in significantly low levels in the product profiles of TaLOG10-1B and TaLOG5-4A2. These results suggested that iPRMP was almost fully consumed to mostly produce iP. TaLOG10-1B and TaLOG5-4A2 from the iP group exhibited extremely low iPR/iP ratios, suggesting their predominant and strong phosphoribohydrolase activity.

**Figure 6:**
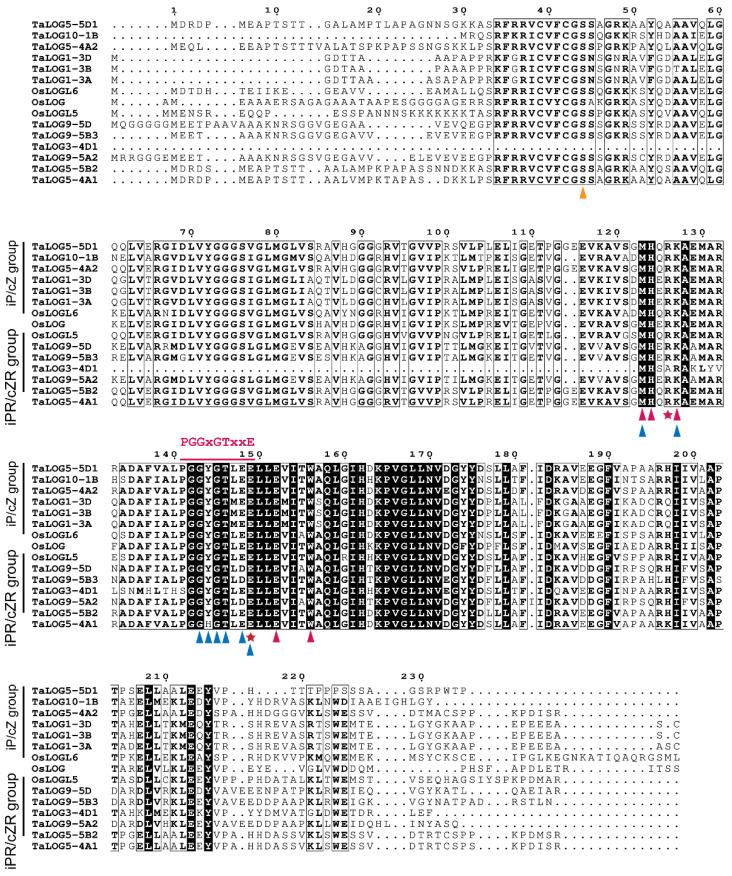
A comparison of residues of LOG proteins with different catalytic activities. Residues involved in catalysis, AMP binding and prenyl group binding are indicated by a red pentagram, blue triangles, and red triangles, respectively. The “PGGXGTXXE” motif is indicated with a red line. The yellow triangle indicates the serine residue for phosphohydrolase activity.

Interestingly, TaLOG5-4A1, TaLOG5-4A2, TaLOG5-5D1, and TaLOG5-5B2, which belong to group V and are closely related to GY3/OsLOGL5 (Figure 2A), displayed distinct enzymatic activities (Figure 5D–E). Specifically, TaLOG5-4A1 and TaLOG5-5B2 primarily produced iPR, while TaLOG5-5D1 and TaLOG5-4A2 primarily produced iP. Taken together, our results demonstrated that the phylogenetic relationship of LOG proteins did not correlate with their enzyme activity. We hypothesized that specific residues might underlie the functional differences between the two groups of LOG proteins. Unfortunately, despite the conservation of known residues involved in substrate binding and catalysis (Figure 6), we were unable to identify clear residue distinctions based on enzymatic group separation. We also performed protein structure analysis by using AlphaFold 3 [31] on the AlphaFold server (https://alphafoldserver.com/, accessed on 13 October 2024). The AlphaFold model produced confident prediction of the central part of all tested LOG proteins, but it struggled to predict the N- and C-terminal regions (Figure S2). With the predicted protein structure, pairwise structure alignments were conducted to explore the potential distinction between these LOG proteins. Apart from the non-reliable N- and C-terminal regions, the highly confident central parts of each comparison overlapped very well (Figure S2). Furthermore, our results showed that all comparisons exhibited low Root Mean Square Deviation (RMSD) values and Template Modeling (TM) scores, approaching 1 in all comparisons (Table 2). Thus, the structure comparison, at least with the protein-only model, still could not give us a clear clue about the enzymatic distinction.

## 4. Discussion

Our phylogenetic analyses of 159 LOG sequences thoroughly revealed the phylogenetic history of the LOG family, and indicated that all land plant LOGs evolved from green algae (Figure 2A). Expansion of LOG members began in moss, since multiple LOG sequences were found in *P. patens* and *S. fallax*, but a single copy of LOG was found in green algae, hornworts, and liverworts (Figure 1 and Figure 2A,B). This is congruent with the large numbers of gene duplications unveiled in the evolutionary past of *P. patens* [32]. Three major genome duplication events occurred before the diversification of vascular plants, which gave rise to four distinct groups of LOG proteins (Figure 2A). A recent study has found that LOG homologs are responsible for the convergent evolutionary appearance of plant prickles in different plant lineages [33,34]. This prickle phenotype was believed to serve adaptive functions in herbivore deterrence, climbing growth, plant competition, and water retention [33]. Thus, the expansion of LOG members may facilitate the colonization and adaptation of plants in diverse conditions.

Comparison of conserved motifs between algae and early land plant bryophytes revealed the de novo evolution of motif 3 in charophyte LOG proteins, followed by the acquisition of motif 8 at the C-terminus during the transition from unicellular algae to land-adapted bryophytes (Figure 4A). These two motifs were retained in more descendant lineages, suggesting their potential role in plant terrestrialization. The single copy of LOG in *C. variabilis* shares the same order of arrangement of motifs 1, 2, 4, 5, 6, and 7 with other LOGs from more diversified plants (Figure 4A,B), which suggests conserved protein sequences across the whole plant lineages. This is consistent with the finding that CvarLOG1 exhibits similar enzymatic activities to that of OsLOG [14,22].

Our most interesting result is that the phylogenetic position of the LOG protein sequence is not correlated with its enzymatic activities’ diversity. For example, TaLOG5-4A1, TaLOG5-4A2, TaLOG5-5B2, and TaLOG5-D1 are grouped closely together in group V in the phylogenetic tree (Figure 2A) and share similar motif compositions and arrangements, except for the lack of motif 8 in TaLOG5-D1 (Figure 4B). However, catalytic activity analysis showed that TaLOG5-4A2 and TaLOG5-D1 primally produced iP and belonged to the iP group, while TaLOG5-4A1 and TaLOG5-5B2 primally produced iPR and belonged to the iPR group (Table 1). Furthermore, they all shared conserved residues in the critical motif for substrate binding and catalytic activity (Figure 6). Structural comparison analysis using AlphaFold 3 failed to provide a clear indication (Figure S2, Table 2) because many enzymes undergo conformational changes upon binding to the substrate [35]. Analysis of the structure of protein–substrate (LOG-iPRMP) complexes may be a promising way to gain insights into the enzymatic differences between these LOG homologs.

## Figures and Tables

**Table 1:** Enzyme activities of investigated LOG proteins.

Protein	Group	Primary Activities	Product Ratios of iPR/iP
TaLOG5-5D1	V	iPRMP→iP	0.27
TaLOG10-1B	III	iPRMP→iP *	0.02
TaLOG5-4A2	V	iPRMP→iP *	0.01
TaLOG1-3D	II	iPRMP→iP	0.27
TaLOG1-3B	II	iPRMP→iP	0.19
TaLOG1-3A	II	iPRMP→iP	0.15
TaLOG9-5D	IV	iPRMP→iPR	3.42
TaLOG9-5B3	IV	iPRMP→iPR	4.2
TaLOG3-4D1	III	iPRMP→iPR	1.28
TaLOG9-5A2	IV	iPRMP→iPR	2.02
TaLOG5-5B2	V	iPRMP→iPR	1.68
TaLOG5-4A1	V	iPRMP→iPR	3.98

* This protein has the dominant enzyme activity of converting iPRMP to iP.

**Table 2:** Comparison of protein structures among TaLOG5-4A1, TaLOG5-4A2, TaLOG5-5B2, and TaLOG5-D1.

Compare Groups	Identity	Aligned Residues	RMSD	TM-Score
TaLOG5-4A1 vs. TaLOG5-4A2	0.91	206	1.72	0.84
TaLOG5-4A1 vs. TaLOG5-5B2	0.86	224	2.06	0.91
TaLOG5-4A1 vs. TaLOG5-5D1	0.91	195	1.79	0.79
TaLOG5-4A2 vs. TaLOG5-5B2	0.87	203	1.85	0.82
TaLOG5-4A2 vs. TaLOG5-5D1	0.86	195	1.71	0.79
TaLOG5-5B2 vs. TaLOG5-5D1	0.91	193	1.51	0.78

RMSD, Root Mean Square Deviation; TM-Score, Template Modeling Score.

## Data Availability

Dataset available on request from the authors.

## Supplementary Materials

The following supporting information can be downloaded at https://www.mdpi.com/article/10.3390/genes15111420/s1: Figure S1: Two clearly separated clades of phylogenetic results of HMM search against the Pfam database with the identifier PF03641; Figure S2: Pairwise structure alignment of LOG proteins; Table S1: Primers used in this study; Table S2: Information of LOG sequences used for phylogenetic tree construction.
