# Supplementary material for: Evolutionary Analysis and Catalytic Function of LOG Proteins in Plants
Source: Genes (Basel). 2024 Oct 31;15(11):1420. doi: 10.3390/genes15111420 (PMC11593424; doi:10.3390/genes15111420)
Supplement: Supplementary file 1 [file genes-15-01420-s001.zip › Supplementary Figure.pdf]

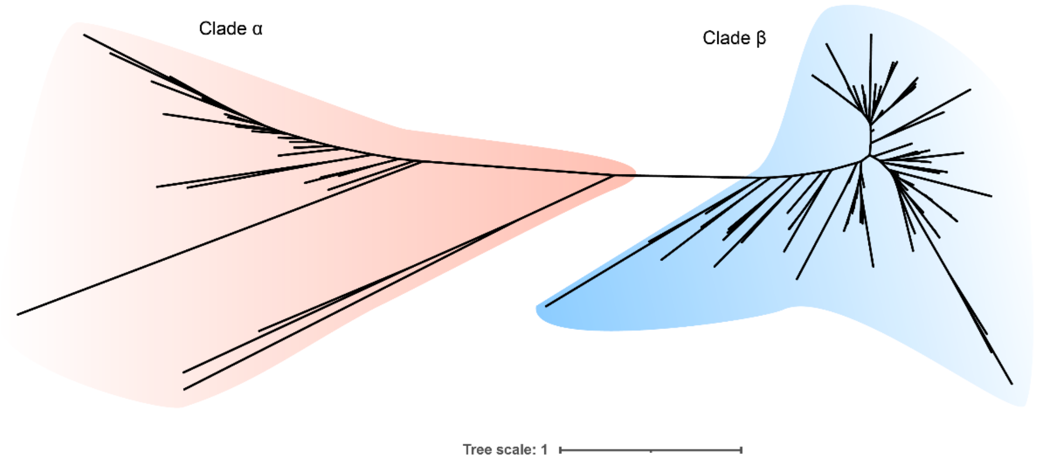

Figure S1. The phylogenetic tree of potential LOG sequences obtained by HMM search and BLASTP methods

Clade α and clade β are separated by a long branch. All of the previous reported LOG proteins are grouped in the clade β.

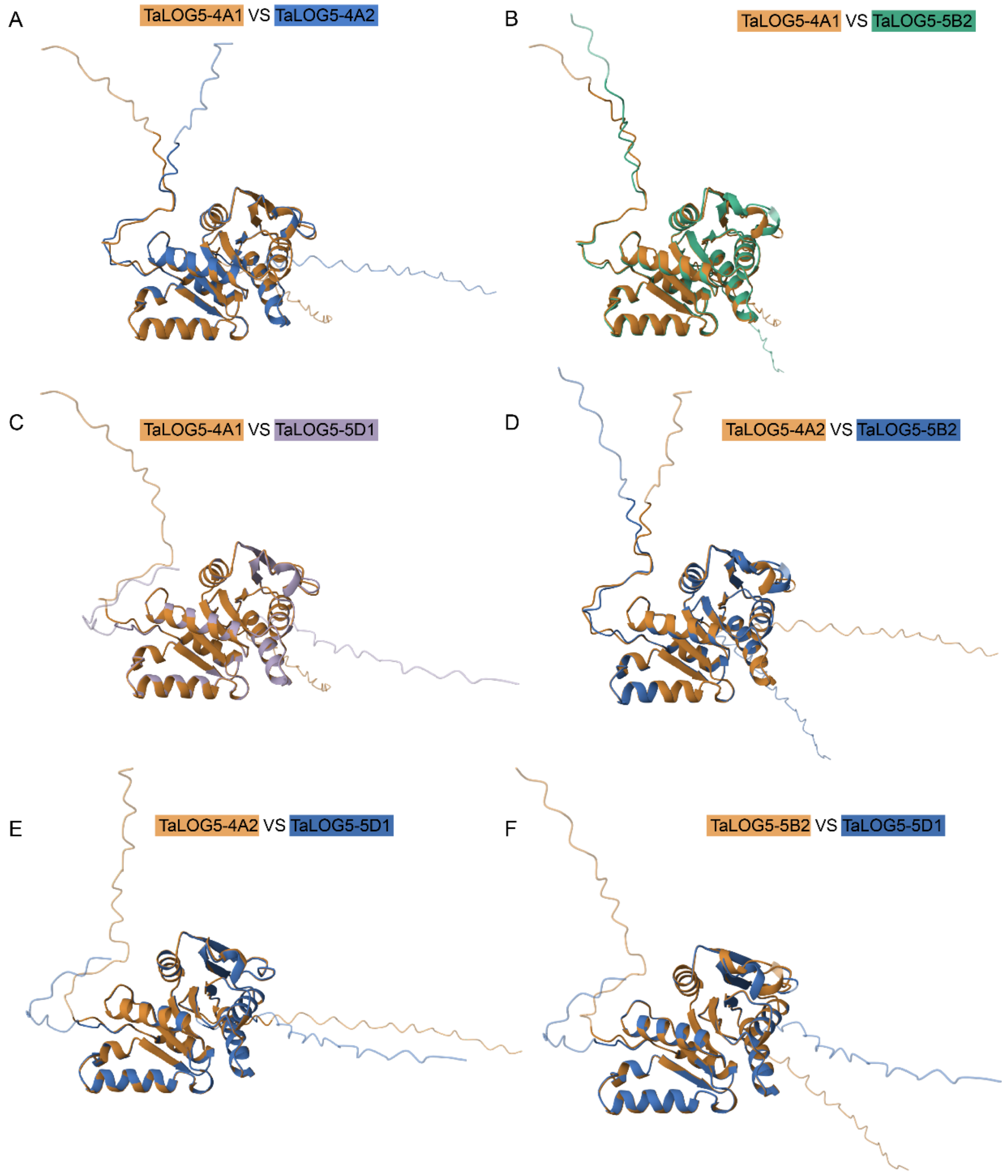

Figure S2. Pairwise structure alignment of LOG proteins
